# Species Distribution and Isolation Frequency of Nontuberculous Mycobacteria, Uruguay

**DOI:** 10.3201/eid2605.191631

**Published:** 2020-05

**Authors:** Gonzalo Greif, Cecilia Coitinho, Jakko van Ingen, Carlos Robello

**Affiliations:** Institut Pasteur Montevideo, Montevideo, Uruguay (G. Greif, C. Robello);; Comisión Honoraria de Lucha Anti-Tuberculosa y Enfermedades Prevalentes, Montevideo (C. Coitinho);; Radboud University Medical Center, Nijmegen, the Netherlands (J. van Ingen);; Universidad de la República, Montevideo (C. Robello)

**Keywords:** Nontuberculous mycobacteria, mycobacteriology, epidemiology, bacteria, NTM, TB, Uruguay, South America, tuberculosis and other mycobacteria

## Abstract

Nontuberculous mycobacteria (NTM) increasingly are recognized as opportunistic pathogens of humans. NTM species distribution is well documented in Europe and North America, but data from other regions are scarce. We assessed NTM isolation frequency and species distribution in Uruguay during 2006–2018.

Nontuberculous mycobacteria (NTM) are comprised of >150 species (*1*) and are increasingly recognized as opportunistic pathogens (*2*). In addition, frequency of disease-causing NTM isolation has been rising in many settings (*3*,*4*). The exact epidemiology of NTM pulmonary disease, the most common manifestation in adults (*3*), has been difficult to determine because reporting is not mandatory in most countries and identification of true disease is often difficult (*5*).

The distribution of NTM species isolated from human clinical samples varies greatly by region (*6*). In South America, data on NTM isolation frequencies are available for São Paulo and Rio de Janeiro, Brazil (*7*–*9*) and Buenos Aires, Argentina (*6*). Because clinical and laboratory observations suggested an emergence of NTM isolates, we conducted a retrospective study of isolation frequency of NTM in Uruguay.

## The Study

We conducted a retrospective study of data on all NTM isolates identified at the Comisión Honoraria de Lucha Anti-Tuberculosis (Montevideo, Uruguay), the national tuberculosis reference laboratory, during January 2006–December 2018. The laboratory receives samples from all suspected tuberculosis (TB) cases across the country and performs universal acid-fast bacillus smear testing and solid culture in Löwenstein-Jensen and Ogawa egg-based media as a part of routine diagnostic testing. We abstracted basic patient demographic information and NTM identification results from the laboratory databases.

Phenotypic characterization of isolated NTM was performed by using biochemical methods, and complementary genotyping identification was introduced in 2012 by using GenoType Mycobacterium CM **(**Hain Lifescience GmbH, https://www.hain-lifescience.de). In 2016, the laboratory included GenoType Mycobacterium AS (Hain Lifescience GmbH) in its pipeline and retrospectively identified previously unidentified isolates.

For comparison, we also collected data on the number of culture-positive TB cases during the study period from the laboratory databases. We used official population information from the National Institute of Statistics Uruguay (Instituto Nacional de Estadística, http://www.ine.gub.uy) to calculate NTM incidence (Appendix 1).

**Figure 1:**
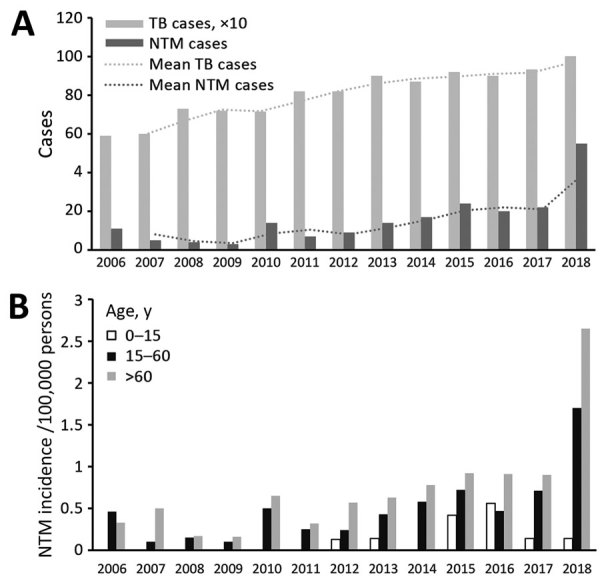
Number of cases of nontuberculous mycobacteria and tuberculosis, Uruguay, 2006–2018. A) NTM and TB cases by year. B) NTM incidence adjusted by age range and year. NTM, nontuberculous mycobacteria; TB, tuberculosis.

During 2006–2018, a total of 255 NTM isolates were collected from pulmonary and extrapulmonary samples from 204 patients, 143 male and 61 female, in Uruguay (Table 1). Most (147/255; 57.6%) isolates identified were members of the *Mycobacterium avium* complex (MAC), which includes *M. intracellulare* and *M. avium*; 21 (8.2%) were *M. kansasii*; 15 were *M. gordonae* (5.9%); and 12 were *M. peregrinum* (4.7%) (Table 2). We observed an increase in NTM isolation frequency and an increase in TB cases during 2011–2018 (Figure 1, panel A). We also calculated an age-adjusted isolation frequency to determine population aging effect (Figure 1, panel B).

**Table 1:** Specimen sources for isolation of nontuberculous mycobacteria, Uruguay*

Specimen source	No. (%)
Pulmonary	
Sputum	170 (66.6)
Bronchoalveolar lavage	18 (7.0)
Bronchoalveolar secretions†	8 (3.1)
Lung biopsy	6 (2.3)
Pleural fluid	3 (1.2)
Ear, nose, throat aspirate	2 (0.8)
Puncture fluid	3 (1.2)
Total	210 (82.3)
Extrapulmonary	
Blood culture	15 (5.9)
Myelocyte culture	9 (3.5)
Skin or soft tissue abscess	4 (1.6)
Urine	1 (0.4)
Feces	2 (0.8)
Gastric lavage	1 (0.4)
Ganglion biopsy	3 (1.2)
Ascites fluid	3 (1.2)
Total	38 (14.9)
Multiple isolation sources‡	3 (1.2)
Missing data	4 (1.6)
*The data include isolates from the same patient over time. †Includes tracheal aspirations. ‡Includes pulmonary and nonpulmonary samples of the same patient.	

**Table 2:** Nontuberculous mycobacteria species isolated in Uruguay, 2006–2018*

Species	2006	2007	2008	2009	2010	2011	2012	2013	2014	2015	2016	2017	2018	Total	% Total
*Mycobacterium intracellulare*	6	5	2	2	4	3	6	3	6	13	9	10	17	86	33.7
*M. avium*	4	0	1	1	7	2	3	2	5	6	3	6	21	61	23.9
*M. kansasii*	0	0	0	1	2	3	2	2	5	1	2	2	1	21	8.2
*M. gordonae*	2	1	0	0	0	0	0	2	4	2	0	2	2	15	5.9
*M. peregrinum*	0	0	0	0	1	1	1	0	1	1	3	2	2	12	4.7
*M. chelonae*	0	0	0	0	0	0	1	1	1	1	2	0	2	8	3.1
*M. fortuitum*	0	0	0	0	0	0	0	2	1	0	1	2	2	8	3.1
NTM, no species determinant	0	0	0	0	0	0	0	0	0	1	3	1	0	5	2.0
*M. genavense*	0	0	0	0	0	0	0	0	0	3	1	0	0	4	1.6
*M. avium* MTBC†	0	0	0	0	0	1	0	2	0	0	0	0	0	3	1.2
*M. intracellulare* MTBC†	0	0	0	0	1	0	0	0	2	0	0	0	0	3	1.2
*M. lentiflavum*	0	0	0	0	0	0	0	0	0	1	0	2	1	4	1.6
*M. scrofulaceum*	0	0	0	0	0	2	0	0	0	0	1	0	1	4	1.6
*M. simiae*	0	1	0	0	0	0	0	0	0	0	2	0	0	3	1.2
*M. abscessus*	0	0	0	1	0	0	1	0	0	0	0	0	1	3	1.2
*M. heckeshornense*	0	0	0	0	0	0	0	1	1	0	0	0	0	2	0.8
*M. xenopi*	0	0	1	0	0	0	0	1	0	0	0	0	0	2	0.8
Other species‡	0	0	0	0	0	0	0	3	0	4	0	1	3	11	4.3
Total	12	7	4	5	15	12	14	19	26	33	27	28	53	255	100.0
*MTBC, *Mycobacterium tuberculosis* complex; NTM, nontuberculous mycobacterium. †Mixed infections. ‡Other species included 1 isolate each of *M. arupense*, *M. asiaticum*, *M. koreense*, *M. shimodei*, *M. interjectum*, *M. marinum* and *M. malmoense*, and mixed infections of *M. avium*–*M. intracellulare*, *M. gordonae*–*M. chelonae*, *M. intracellulare*, *M. fortuitum*, *M. intracellulare*-MTBC.															

Among 204 cases, 23 patients had >1 NTM-positive isolate 6 months apart, and 13 had positive cultures for >2 years. Patients >50 years of age (mean 52 years of age) were more likely to have prolonged culture-positivity, a statistically significant difference from patients with a single positive culture (mean 42.6 years of age; p = 0.0099). Most (7/13; 53.8%) prolonged infections were caused by *M. intracellulare*, and 4 prolonged cases remained NTM-positive for <6 years, including 2 infections with *M. intracellulare* and 1 each with *M. kansasii* and *M. heckeshornense.* Most (10/13; 76.9%) prolonged cases were detected in pulmonary isolates.

Only 4 (1.9%) cases showed disseminated infections in isolates obtained from hemoculture, bone marrow culture, ganglion biopsy, feces, urine, or sputum of the same patient. Among the 4 disseminated infections, *M. avium* was isolated from 2 cases, *M. genavense* from 1 case, and *M. intracellulare* from 1 case.

**Figure 2:**
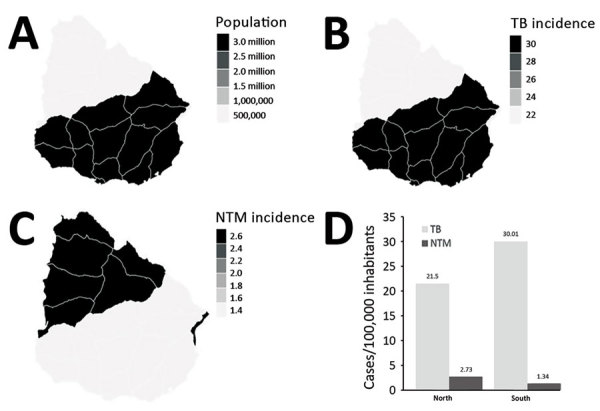
Distribution of cases of tuberculosis and nontuberculous mycobacteria by region and population density in 2018, Uruguay. A) Population density of north versus south regions of Uruguay. B) Incidence of TB cases in 2018. C) Incidence of NTM cases in 2018. D) TB and NTM incidence by region. Even though the population density in the north is much lower than that in the south, the north has a higher incidence of NTM than the south. NTM, nontuberculous mycobacteria; TB, tuberculosis.

The incidence of NTM in Uruguay increased from 0.33 cases/100,000 inhabitants in 2006 to 1.57 cases/100,000 inhabitants to 2018 (Figure 1, panel A). In 2018, the incidence of NTM was 2.73/100,000 inhabitants in the north, which has only 16.72% of the total population of the country. In the south, where 83.28% of the population lives, the incidence was much lower, 1.34 cases/100,000 inhabitants. Culture-positive TB cases showed a reverse tendency with statistically significant differences. The incidence rate for TB was higher in the south, 30.01 cases/100,000 inhabitants, and lower in the north, 21.50 cases/100,000 inhabitants (Figure 2).

During the period studied, we observed a 4-fold increase in the NTM isolation rate (Appendix 1). Implementation of new detection techniques during this timeframe could account for part of the increase (*10*). However, we noted a >2-fold increase (2.49) in number of isolates recorded during 2017–2018, when no laboratory protocol changes were introduced, suggesting the NTM incidence is rising in Uruguay (Appendix 1). 

Rivero-Lezcano et al. (*10*) suggested changes in the pathogen and an aging population could explain the rising incidence of NTM isolation and disease. From 2017 to 2018, we observed a higher increase in NTM incidence (2.94 cases/100,000 persons) among persons >60 years of age compared with persons 15–60 years of age (2.39 cases/100,000 persons) (Figure 1, panel B). However, we noted that the age composition of the population remained stable over time (Appendix 1), suggesting other factors are driving increases in NTM isolation rates.

MAC is reported to be the most frequently isolated NTM species in all continents (*3*), but we observed a different species distribution than previously reported for South America. We saw a higher prevalence of *M. intracellulare* (33.7% of isolates) than *M. avium* (23.9% of isolates) (Table 2). We found species not previously reported in Latin America, including *M. xenopi* and *M. arupense*, and *M. malmoense*, which typically is found in northern Europe (*11*). We also found *M. parakoreense*, for which only 2 cases are reported in the literature, 1 case in 2013 isolated from a single clinical sample (*12*) and another in Africa (*13*). In addition, we noted 2 cases of *M. heckeshornense*, a species previously reported by our group (*14*).

We noted prolonged positivity in 6% (13/204) of NTM cases. MAC was responsible for most (69%) prolonged culture positives and *M. kansasii* was responsible for 15% of prolonged positivity.

We believe the differences observed in NTM isolation rates between regions of Uruguay could be explained by climate factors. The higher temperatures and increased rainfall in the north could favor the incidence ambient pathogens (Appendix 2). We also cannot disregard differences associated with other factors, such as the age of the population.

Our study has several limitations. First, the public health system of Uruguay only registers *M. tuberculosis*; clinical data of NTM patients are difficult to obtain. In addition, the GenoType AS assay used to identify NTM does not detect all NTM species; including genomic sequencing-based identification methods would improve detection in the future. Identification of NTM species is crucial for determining appropriate clinical and antimicrobial treatment for pulmonary NTM, which are dependent on the characteristics of the species (*8*).

## Conclusions

Our findings indicate that rate of NTM have risen nearly 5-fold (4.79) in Uruguay, from 0.33 cases/100,000 inhabitants in 2006 to 1.57 cases/100,000 inhabitants in 2018. The species distribution largely aligns with existing data from elsewhere in South America, which demonstrates a predominance of MAC. Prolonged culture positivity and various isolation sites sources suggest that NTM disease is prevalent in Uruguay and warrants further studies to optimize diagnosis and treatment.

Appendix 1 Incidence of nontuberculous mycobacteria isolated among different age groups in Uruguay, 2006–2018.

Appendix 2 Additional information on species distribution and isolation frequency of nontuberculous mycobacteria, Uruguay.
