# Supplementary material for: Species Distribution and Isolation Frequency of Nontuberculous Mycobacteria, Uruguay
Source: Emerg Infect Dis. 2020 May;26(5):1014–8. doi: 10.3201/eid2605.191631 (PMC7181928; doi:10.3201/eid2605.191631)
Supplement: Appendix 2 — Additional information on species distribution and isolation frequency of nontuberculous mycobacteria, Uruguay. [file 19-1631-Techapp-s2.pdf]

Article DOI: https://doi.org/10.3201/eid2605.191631

Species Distribution and Isolation Frequency of Nontuberculous Mycobacteria, Uruguay

Appendix 2

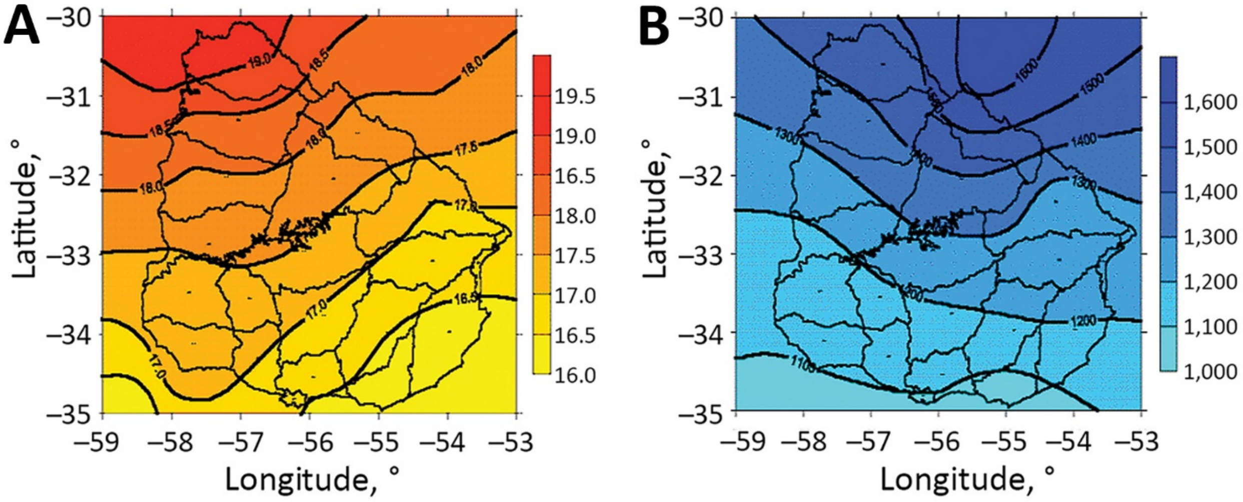

Appendix 2 Figure. Mean temperature and rainfall registered by region, Uruguay, 1961–1990. A) Mean annual temperature; scale bar indicates °C. B). Mean annual rainfall; scale bar indicates mm. Map source, Instituto Uruguayo Meteorología (https://www.inumet.gub.uy).
